# AI Enabled Bridge Bidding Supporting Interactive Visualization

**DOI:** 10.3390/s22051877

**Published:** 2022-02-27

**Authors:** Xiaoyu Zhang, Wei Liu, Linhui Lou, Fangchun Yang

**Affiliations:** 1 School of Computer Science (National Pilot Software Engineering School), Beijing University of Posts and Telecommunications, Beijing 100876, China; xy_zhang@bupt.edu.cn (X.Z.); loulinhui.shey@bytedance.com (L.L.); fcyang@bupt.edu.cn (F.Y.); 2 State Key Laboratory of Networking and Switching Technology, Beijing University of Posts and Telecommunications, Beijing 100876, China

**Keywords:** bridge bidding, imperfect information, deep neural network, interactive visualization

## Abstract

With the fast progress in perfect information game problems such as AI chess and AI Go, researchers have turned to imperfect information game problems, including Texas Hold’em and Bridge. Bridge is one of the most challenging card games that have significant research value. Bridge playing is divided into two phases: bidding and playing. This paper focuses on bridge bidding and proposes a bridge bidding service framework using deep neural networks, and supports bidding visualization for the first time. The framework consists of two parts: the bidding model (BM) with a multilayer neural network, and a visualization system. The framework predicts not only reasonable bids from the existing bidding system of humans, but also provides intuitive explanations for decisions to enable human–computer information interaction. Experimental results show that this bidding AI outperforms majority of existing systems.

## 1. Introduction

Computational games have been important research to verify the development level of artificial intelligence. At present, Artificial Intelligence has seen several breakthroughs in perfect information games. Chess [1] and Go [2,3,4] are typical representatives in which players can obtain the whole state of games. The search space of chess can realize a global search under the current computing resources and find the perfect solution with computing power to defeat human beings. DEEPMIND has made a breakthrough in the research of Go. The AlphaGo-Zero algorithm does not require inputting human knowledge and can update the neural network through self-play, achieving the effect of defeating human experts [4]. Recently, computer programs defeated humans in 2-player and 6-player no-limit Texas Hold’em [5,6,7,8,9].

Nevertheless, imperfect information games such as Mahjong [10], DeltaDou [11,12], and Bridge, where players cannot obtain the whole state of games, are still very challenging. Existing methods for resolving perfect information games do not directly apply to imperfect information games. However, most decision-making problems in the real world are under imperfect information, so it is necessary to study the imperfect information games. Hence, this paper focuses on a typical imperfect information game—Bridge.

Bridge uses standard 52 cards to compete, and the four players are usually considered East, West, South, and North. East–West and South–North teams confront, and finally get high scores. Bridge is mainly divided into two phases: bidding and playing. In the bidding phase, four players from two teams use intra-team collaboration and inter-team confrontation to transmit hand information through bidding to determine the final contract, which influences the final score. The four players each hold a random 13 cards and can only see their own 13 cards. There are $6.35\times 10^{11}$ possible hand holdings [13]. Then, players used 38 bids to bid in clockwise order. All bids are divided into three non-bids (PASS, DOUBLE, and REDOUBLE) and a set of orderly bids: { 1♣, 1♢, 1♡, 1♠, 1NT, 2♣, …, 7NT }. The PASS means that the player skips this round of bidding; the DOUBLE or REDOUBLE means that the player’s score needs to be doubled. The bidding proceeds until it is terminated by three consecutive PASS, at which time the last bid is regarded as the contract of the bidding. The contract consists of two parts: a number and a symbol. The number indicates that the winning team needs to win at least additional rounds to complete the contract during the subsequent playing stage, and the symbol indicates the trump suit.

In the playing phase, the four players play a card in clockwise order. A total of 13 rounds of cards will be played. The players use certain skills to win as many rounds as possible, trying to complete their contract or prevent the opponent from completing the contract. Although several algorithmic techniques have been successfully applied to the playing phase, bidding needs a different approach. This paper focuses on Bridge bidding.

Bridge bidding can be divided into two sub-problems: the bidding without competition [14,15,16] and the bidding with competition [17]. The sub-problem of the bidding without competition assumes that the opponent always calls PASS when bidding, so the exchange of information between teammates will not be prevented. Nevertheless, this situation does not conform to the real competition. Hence, we focus on the sub-problem of the bidding with competition, which means that each AI player may bid and exchange information as in real bridge games. The main contributions of our work are summarized as follows:

- We trained a bidding model (BM) based on deep neural networks, which can learn the human bidding system with only expert data and no manual input of rules. It can achieve human-machine competition.
- We designed a visualization system that can help beginners learn more rules in the bidding system.

The rest of this paper is structured as follows. Section 2 discusses related work. Section 3 proposes the model framework and describes the visualization system in detail. This whole project is hosted at https://youtu.be/x0VVyg8C2i4 (accessed on 1 October 2020). Then we introduce experimental setups and present the results with evaluation in Section 4. Finally, we conclude our work in Section 5.

## 2. Related Work

The research on Bridge began in the 1980s. In the first World Computer Bridge Championship in 1997, the program Bridge Baron [18], written by Tom Throop, won the championship. In the second year, the GIB program developed by Matthew Ginsberg won the championship [19]. In the following years, Jack and Wbridge5 http://www.wbridge5.com/ (accessed on 1 October 2020) took turns to win the championship [20]. These traditional Bridge AIs have manually input complicated bidding rules into computer programs. However, since the number of the possible bidding sequences (see the analysis at http://tedmuller.us/Bridge/Esoterica/CountingBridgeAuctions.htm (accessed on 1 October 2020)) is as high as $10^{47}$, it is hard for these human-designed rules to cover all the situations, which leads to ambiguous bidding rules. In addition, there may be multiple conflicting interpretations for a bid. Human players typically spend much time practicing with their peers to improve mutual understanding and minimize bids’ ambiguity. However, it is highly challenging for AI players to resolve ambiguity by replicating human bidding systems to achieve the same mutual understanding that human players can achieve, which puts them at a disadvantage in the bidding phase.

In order to overcome the shortcomings mentioned above, some researchers have made relevant attempts to improve the human bidding system. For example, Amit et al. [13] proposed a Monte Carlo sampling approach based on the human bidding system to solve the ambiguity of bidding by building a decision tree model. DeLooze and Downey [21] used a human bidding model to generate a large amount of training data and clustered the bidding process utilizing self-organizing mapping for ambiguity resolution. Nevertheless, this model can only be used to bid no trump hands effectively. Human bidding systems play a central role in AI players’ bidding in those works. Ho and Lin [14] proposed a learning framework based on the UCB algorithm that did not rely on the human bidding system, which demonstrated the possibility to learn to bid directly in a data-driven manner. Unfortunately, the study is somewhat limited by the decision tree model with a limit of up to five choices per bid, which does not correspond to the actual bidding situation. Yeh and Lin [15] first tried using deep reinforcement learning to bid without depending on any human expert knowledge but did not consider the bidding with the competition. In [17] and [22], they all begin to focus on the sub-problem of the bidding with competition.

We find that decision making has been applied in various fields like economics [23] and robot applications [24]. There are many papers using different techniques, such as machine learning and deep learning, for decision making. Most of the above Bridge bidding algorithms and models assist players in making decisions, but they are difficult to explain the basis behind the decision. Therefore, we extend some ideas from [22] and propose an AI bidding framework that can not only predict bids but also provide intuitive explanations for decisions made to help human–computer information interaction.

## 3. Framework

**Figure 1:**
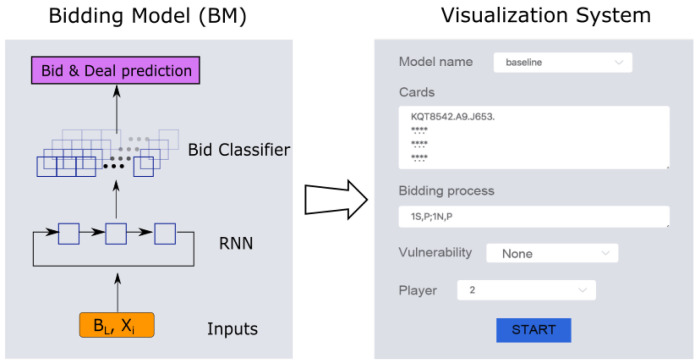
An overview of the proposed framework. It includes two parts: the bidding model and the visualization system.

In this section, we introduce the details of our proposed framework, shown in Figure 1. The framework includes the bidding model(BM) and a visualization system. BM can predict the probability distribution of bids under the natural bidding system and output the characteristics conveyed by other players’ bids in the current bidding process. The visualization system can more intuitively display the prediction results of the characteristics of other players’ hands.

### 3.1. Bidding Model (BM)

**Figure 2:**
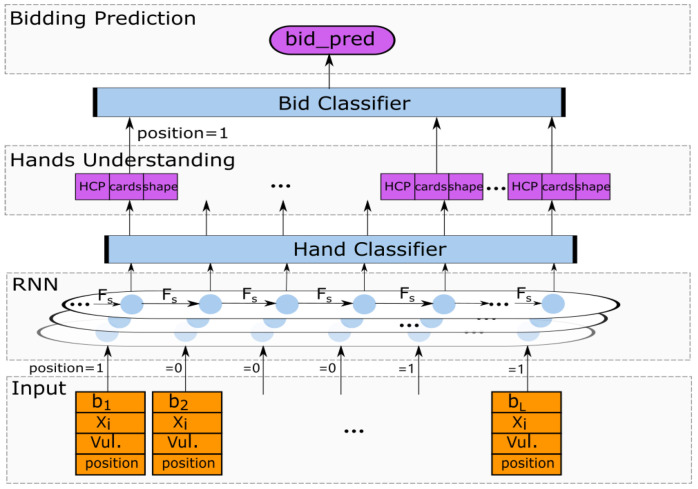
The structure for BM.

Bridge is a game of teamwork in which two players of a partnership use the same bidding system to exchange information. The bidding system is composed of many rules that give each player the meaning of every possible bid, which is equivalent to providing a communication language that allows players to use bids to exchange information about the cards they hold. Therefore, we use 4 million pairs of real bidding data provided by the Synrey platform for human–machine confrontation under the natural bidding system to train a bridge bidding model. The model is based on expert experience that supports the natural bidding system. Obviously, a single bid cannot predict the next bid in the bidding phase. The current historical bidding sequence can directly affect the next bidding. Hence, we choose Recurrent Neural Network (RNN) as the main body of our model. The specific structure of the BM is shown in Figure 2, which consists of two parts, one is a bidding prediction system based on imitation learning, and the other is a visualization system for hands understanding. This visualization system can help players transfer hand information to each other and evaluate the value of the hands to help predict bidding more accurately.

BM contains a three-layer stacked Recurrent Neural Network(RNN) and multi-layer fully connected neural network. To facilitate the description, we first define some symbols shown in Table 1. The input layer accepts the player *i*’s hands $X_{i}$, bidding action $b_{L}$, vulnerability *v* and position *p*. After getting the embedding vectors, we concatenate them to be the input of a three-layer stacked RNN. The hidden state *s* is finally encoded by the RNN, which is as follows:

(1) $$\begin{array}{cc} \; & s=F_{s}\left(X_{i},B_{L}\right),\\ \; & B_{L}=\left\{b_{L},v,p\right\},\\ \; & i=1,2,3,4;\;L=\{1,2,\cdots ,n\}, \end{array}$$

where $F_{s}\left(•\right)$ is a recursively called nonlinear activation function of the recurrent neural network. $B_{L}$ represents the *L*th bid in a bidding sequence.

Simultaneously, the outputs of the hidden states encoded by RNN are fed into the multi-layer fully connected neural network, i.e., MLP (Multi-Layer Perception). We then define function $F_{mlp}^{H}\left(•\right),F_{mlp}^{C^{suit}}\left(•\right)$, $F_{mlp}^{Y}\left(•\right)$ and $F_{mlp}^{G}\left(•\right)$ for four types of MLP units. The overall formula in features of external information are as follows.

(2) $$\begin{array}{cc} \; & s_{H}=F_{mlp}^{H}\left(s\right),\\ & s_{C^{suit}}=F_{mlp}^{C^{suit}}\left(s\right),\\ & s_{Y}=F_{mlp}^{Y}\left(s\right),\\ \; & s_{G}=F_{mlp}^{G}\left(s,s_{H},s_{C^{suit}},s_{Y}\right)\times P_{mask}^{B}\left(L\right), \end{array}$$

where suit={♣,♢,♡,♠}.

In order to prevent the violation of bridge rules in the bidding prediction, we set up a rule filter vector $P_{mask}^{B}$. When a bid violates the bridge rules in a certain state, we define the weight of such bid to 0 so that the rule of Bridge can be gradually learned. We define $P_{mask}^{B}\left(L\right)$ as follows:

(3) $$P_{mask}^{B}\left(L\right)=\left\{\begin{array}{cc} 1 & \mathrm{the}\;L\mathrm{th}\;\mathrm{bid}\;\mathrm{is}\;\mathrm{legal},\\ 0 & \mathrm{the}\;L\mathrm{th}\;\mathrm{bid}\;\mathrm{is}\;\mathrm{illegal}. \end{array}\right.$$

We will verify the importance of the rule vector in Section 4.2.

The output layer of BM consists of the hands understanding and the bidding prediction. The hands understanding system has three outputs: the probability distribution of the HCPs of the teammate $P(H=h|X_{i},B_{L})$, the probability distribution of the number of cards of each suit $P(C^{suit}=c|X_{i},B_{L})$ and the probability distribution of the hands of the other three players $P(Y_{i}^{p}=1|X_{i},B_{L})$. High Card Points (HCPs) are total points of a full deck of cards. A = 4, K = 3, Q = 2, J = 1. The total number of HCPs is 40, and the expected number of HCPs in the given hands is 10.

(4) $$\begin{array}{cc} P(H=h|X_{i},B_{L}) & =P(H=h|s_{H})\\ \; & =softmax(w_{h},s_{H})\\ \; & =\frac{exp(w_{h},s_{H})}{\sum_{h=0}^{37}exp\left(w_{h},s_{H}\right)},\\ P(C^{suit}=c|X_{i},B_{L}) & =P(C^{suit}=c|s_{c^{suit}})\\ \; & =softmax(w_{c^{suit}},s_{c^{suit}}),\\ \; & =\frac{exp(w_{c^{suit}},s_{c^{suit}})}{\sum exp(\left(w_{c^{suit}},s_{c^{suit}}\right))},\\ P(Y_{i}^{p}=1|X_{i},B_{L}) & =P(Y_{i}^{p}=1|s_{Y})\\ \; & =\frac{exp(w_{i}^{p},s_{Y})}{1+exp(w_{i}^{p},s_{Y})}, \end{array}$$

By combining Equations (1), (2) and (4), we get the objective function *I* of the hand understanding as the manually specified feature.

(5) $$\begin{array}{cc} I(X_{i},B_{L}) & =P(H,C,Y|X_{i},B_{L})\\ \; & =\{P\left(H|X_{i},B_{L}\right),P\left(C|X_{i},B_{L}\right),P\left(Y|X_{i},B_{L}\right)\} \end{array}$$

Note that the primary purpose of bridge bidding is to find the best possible contract and convey some information to partners. So, the bidding prediction system describes a bid probability distribution that conforms to the rules of the system based on the features coded by the model and the manually specified feature, in which the objective function *G* is as follows:

(6) $$\begin{array}{c} \begin{array}{cc} G(X_{i},B_{L},I) & =P(b_{L+1}=\alpha |X_{i},B_{L},I)\\ \; & =P(b_{L+1})\\ \; & =softmax(w_{G},s_{G})\\ \; & =\frac{exp(w_{G},s_{G})}{\sum_{h=0}^{37}exp\left(w_{G},s_{G}\right)}. \end{array} \end{array}$$

**Figure 3:**
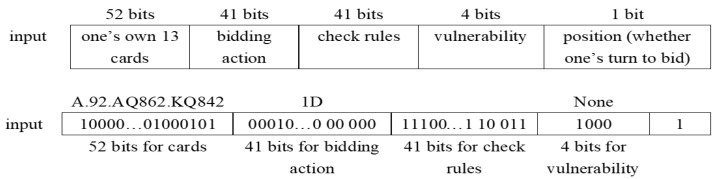
An example of model input.

**Figure 4:**
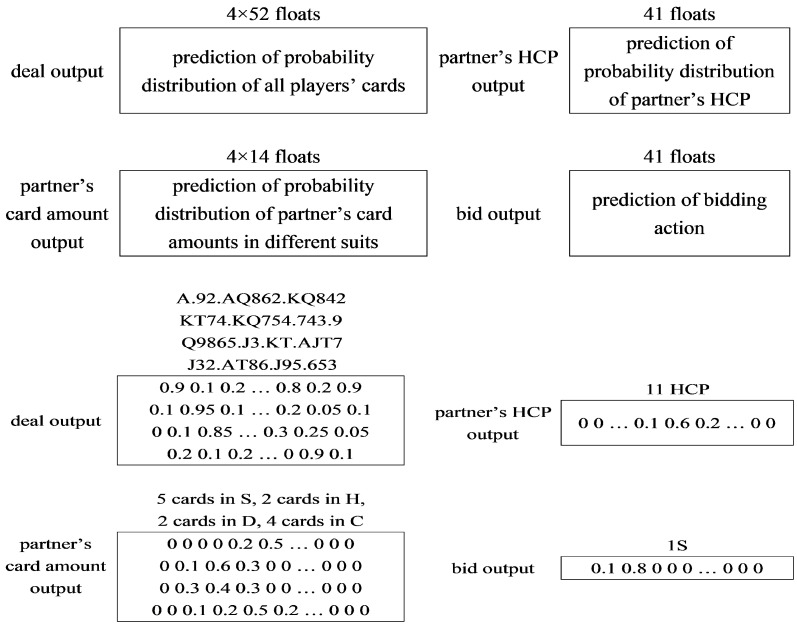
An example of model output.

Figure 3 and Figure 4 respectively show examples of model input and output. We can see that the model’s input is the player’s hands, bidding action, check rules, vulnerability, and position, followed by three-layer RNN. A 52-dimensional vector represents the player’s hands, and a 41-dimensional vector is used to check rules that can ensure the bidding sequence’s rationality. We also add the vulnerability to the input for better score calculation. Besides this, one of the most important factors is the position, which means whether one’s turn to bid. The importance of this factor will be demonstrated in the experiment later.

### 3.2. Visualization System

**Figure 5:**
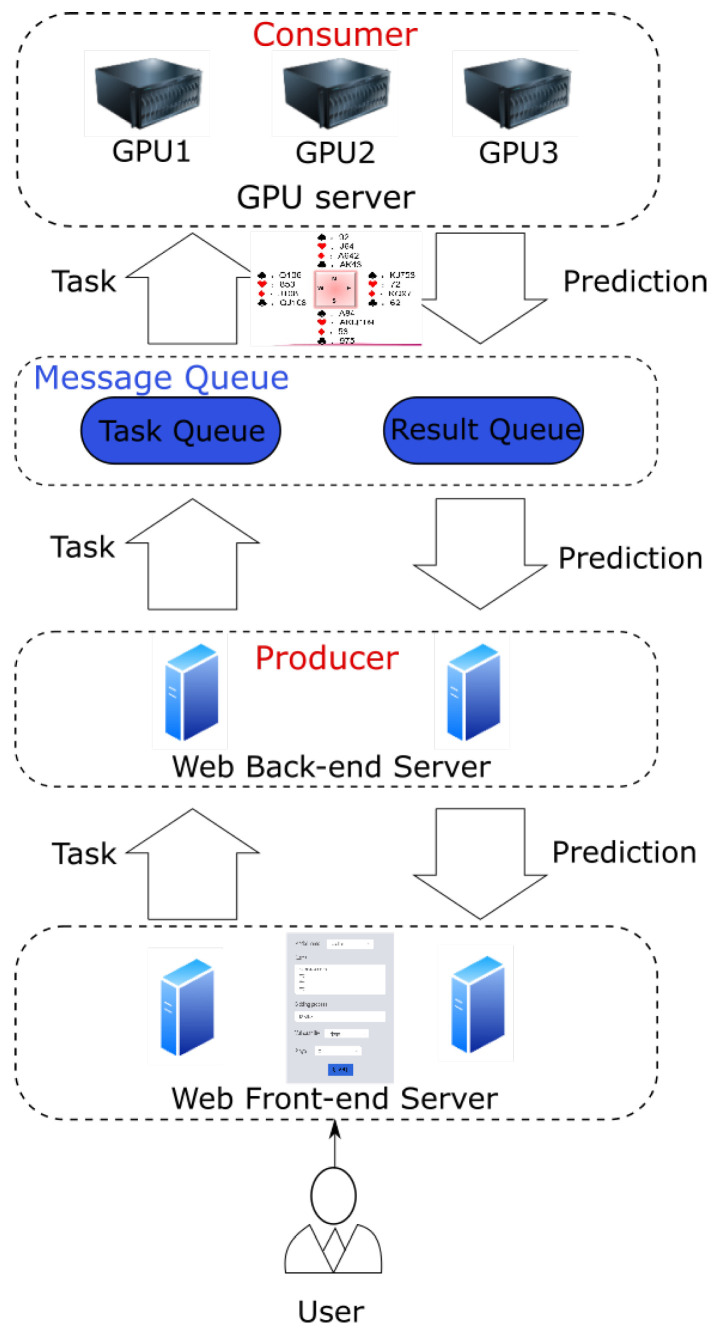
General architecture of visualization system.

According to the rules of Bridge, players have the right to let their opponents explain the meaning of their current bids. For human players, explaining their decisions is very simple, but in a human–computer game, how do the robots explain the meaning of their decisions to humans. Figure 5 is a visualization system framework we designed, which is mainly divided into three parts: web front-end server, web back-end server, and GPU server. The user first accesses the visual page in the web front-end server, enters the player’s own hands and the current bidding process, and sends the task to the web back-end server. The web back-end server sends the task to the task queue, and all GPU nodes will process the task after competitively obtaining the task. Then, after the GPU node obtains the prediction result, it stores the result in the result queue, and finally, the web back-end server will get the corresponding result from the result queue and send it to the front-end server to present it to the user.

**Figure 6:**
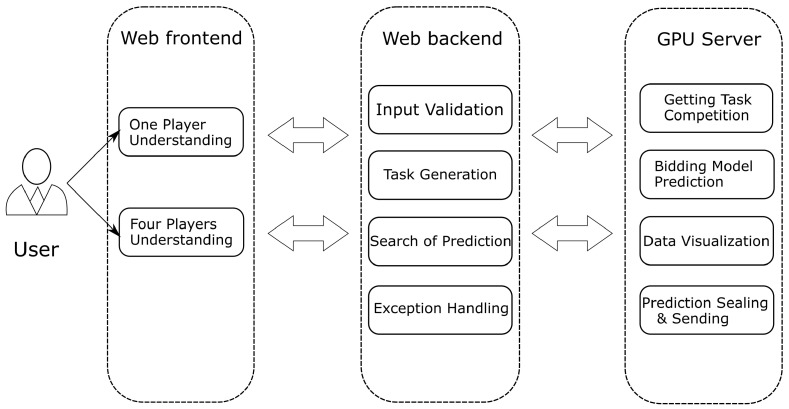
The specific functions of each part.

The web front-end provides a user interface to facilitate sending user tasks to the web back-end server, and at the same time, it can display the final returned results to the user. The web back-end server needs to provide APIs for the web front-end to call. It is responsible for interacting with the GPU server and sending tasks input by the user to the GPU server. The bidding model on the GPU server makes predictions and returns the prediction results to the web back-end. The web back-end server not only needs to be able to perform a simple verification of user input but also has a specific error handling mechanism to deal with the situation where the GPU server does not respond. The specific functions of each part are shown in Figure 6.

#### 3.2.1. Web Back-End Server

The web back-end server is an intermediate module that interacts with the GPU prediction node and the web front-end server. After getting a task, the server will first verify the validity of the data. After the verification is completed, the web back-end server will generate a universally unique identifier for each task to mark it. It is mainly responsible for verifying and encapsulating the user input data. The data with the wrong format will directly return the error message and then send the data to the task queue. The data in the result queue is sent to the web front end for display and then put in the task queue. After being put into the task queue, the server will immediately start polling the result queue to find whether the prediction result corresponding to the task ID appears in the result queue until it times out or gets the task’s predicted result. If it times out, it will return the task timeout error message. If the prediction result is obtained, the corresponding prediction result is returned.

Since the web server needs to obtain the predicted results from the result queue through polling when querying, each query is synchronized and time-consuming. Because the Django framework is single-threaded, it does not support simultaneous processing of multiple requests, so a single synchronous request takes too long to cause other requests to accumulate, and even requests are accumulated on the web server, causing a large number of GPU nodes to become idle. This article chooses to use uwsgi [25] to deploy the Django project so that Django supports multiple processes to process multiple requests at the same time so that the web server can process multiple requests at the same time and improve the resource utilization of GPU nodes.

#### 3.2.2. GPU Server

The GPU server is the system’s core module that uses the deep learning model to make predictions. It consists of model loading, processing prediction tasks, and data visualization. Because of the enormous computing power required, it needs to support a flexible horizontal expansion method. Since most deep learning frameworks do not support multithreading and multi-process, in order to ensure the flexibility of deployment, the system adopts the following two design methods:

1 The GPU server comprises GPU prediction nodes one by one. Each node corresponds to a unique GPU determined when the node is started. It will not change until the node is shut down. If the GPU memory capacity allows, each GPU is bound to any number of nodes. The above model (BM) is loaded on each GPU node to perform bid prediction tasks. This one-to-many relationship between GPU and GPU nodes has the following two advantages:
  - This allows a single GPU to run prediction tasks on multiple nodes simultaneously, which effectively improves GPU computing performance.
  - The one-to-one architecture only executes one prediction task at a time. If this prediction task takes too much time, it is likely to block the subsequent prediction tasks, causing many tasks to accumulate in the message queue. Therefore, the one-to-many architecture alleviates the blocking mentioned above.
2 The web server and GPU nodes do not interact directly through APIs, but they are decoupled through message queues to form a producer-consumer model, which brings the following advantages:
  - The GPU nodes and the web back-end server are completely decoupled, and the services on both sides can be deployed and expanded freely.
  - The distributed nature of the system is stronger and effectively reduces the accumulation of tasks in the message queue by increasing the number of consumers.
  - Ensuring the reliability of the system. If the consumer nodes restart, the failed messages will stay in the message queue instead of disappearing directly, which effectively enhances the system’s reliability.

## 4. Experiment

In this part, we conducted a series of experiments to evaluate our AI. We first verified the reliability of the experimental data. Next, we evaluated the performance of BM in detail and showed the results of the visualization system with examples. Finally, we compared the proposed model with a state-of-the-art model and a well-known computer bridge software, Wbridge5, which won the computer bridge championship for several years.

### 4.1. Dataset

**Figure 7:**
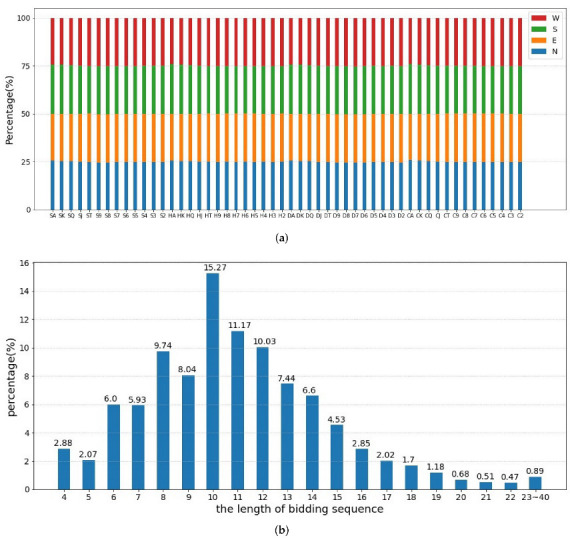
Dataset Evaluation. (**a**) The percentage of each card among four players. (**b**) The distribution of lengths of bidding sequences.

In the experiment, we used the Snyrey platform robot to generate a large amount of club-level data in the natural bidding system. Currently, the Snyrey platform has accumulated nearly 500 million historical bidding records and playing with double-dummy hands. We randomly generated nearly 4,000,000 instances of real bidding as our dataset, 70% of which are used as training data for BM, 20% of which as a validation set, and the remaining data as testing data. Each instance contains the real hands of four players, the historical bidding sequence, and the double-dummy hands’ results. Figure 7a shows that the probability of each card in each player is roughly the same. The distribution of lengths of bidding sequences in the data set are depicted in Figure 7b, which presents a normal distribution. Most of the bids are concentrated at the end of 8∼13, and the probability of too long or short bidding sequences is very low.

### 4.2. Performance Analysis of BM

**Figure 8:**
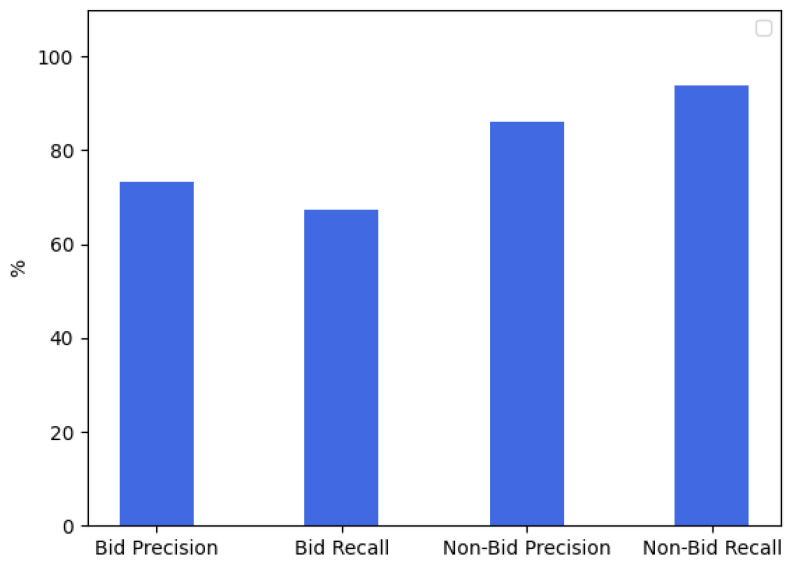
Performance Analysis of BM.

We designed BM based on deep neural networks. Our models were run on a single GPU. We run all the models up to 200,000 epochs. We selected accuracy, precision, and recall as the evaluation criteria to analyze the predicted results of the bid and the non-bid. As shown in Figure 8, the precision of the bid is higher than the recall, and the recall of the non-bid is higher than the precision. It can be seen that the predicted results of the model are more biased towards predicting non-bid. This is because there are fewer types of non-bid in the training dataset while there are more types of bids.

**Figure 9:**
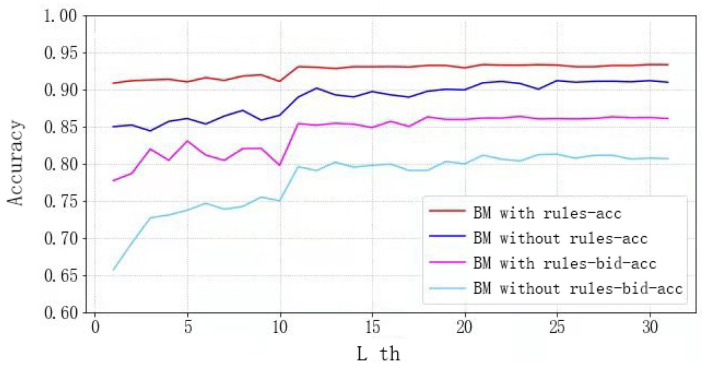
Accuracy of different structures.

In Section 3.1, we designed a rule vector $P_{mask}^{B}\left(L\right)$ to ensure the legitimacy of the output. Next, we verify whether the rule vector affects all bids and bids under the condition that the training data, testing data, and other parts of the model remain unchanged. The experimental results are shown in Figure 9. Since PASS (one of the non-bid) accounts for a large proportion of all bids, in addition to the overall accuracy of all bids, the accuracy of bids (except non-bid) is also added to the evaluation criteria at this stage. It is not difficult to see that, whether it is the overall accuracy of bids or the accuracy of bids (except non-bid), the results of the models with the rule vector are all better than the results of the models without the rule vector. Meanwhile, the model that adds the rule vector converges faster. In a word, the overall accuracy of our model reached 89%.

### 4.3. The Evaluation of Visualization System

The existing bridge AI programs cannot explain the meaning of their bids. In order to better match the real bridge game and realize the real human–computer interaction, we have made a visualization system that can reflect the distribution of bid prediction, player hands, HCPs, and the number of the card—taking an example to illustrate it. We select a representative bidding process to analyze and verify the visualized results and evaluate whether the visualized results can correctly express BM’s understanding. For ease of presentation, in the analysis of experimental results, C, D, H, S are uniformly used to represent suits ♣, ♢, ♡, ♠, and N represents no-trump card. The hands of each player in the test case were represented by a string separated by dots representing the cards of the suits ♠, ♢, ♡, ♣ held by a player.

**Figure 10:**
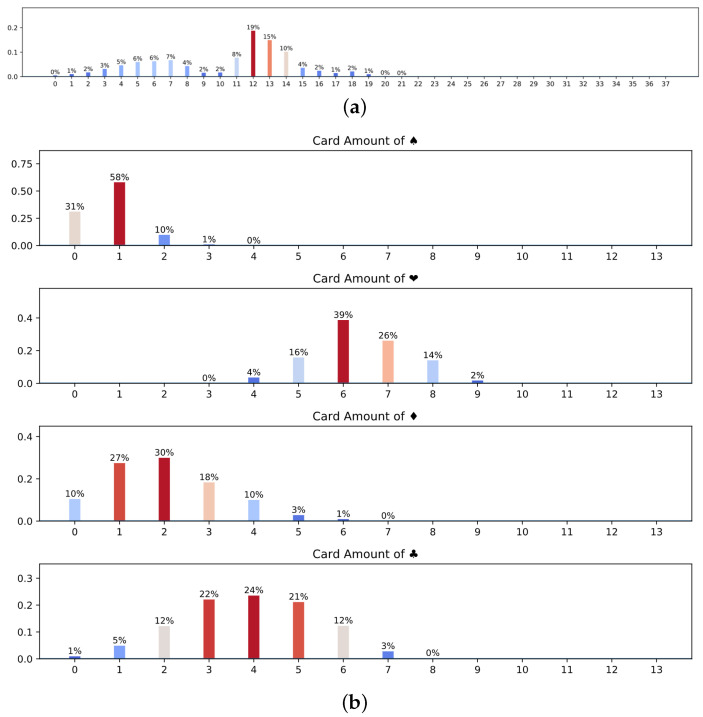

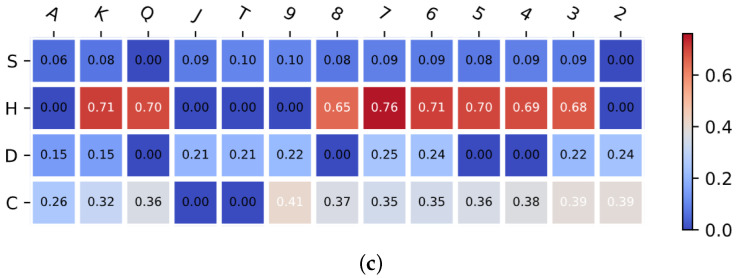
The visualization system. (**a**) Average HCPs of North player; (**b**) The distribution of the number of cards in different suits of West players; (**c**) The distribution of West player’s hands. We show the hands distribution in the form of a heat map. The horizontal axis of the heat map represents the size of the cards, and the vertical axis represents the suit of the cards. The darker the color, the higher the probability of owning the card in hand. It is evident that the West player has more cards in the suit of H(♡).

Table 2 shows that the North player first opened 1D. In the natural system, 1D is an open-bid, which means that the HCPs exceed 12 points and have four cards with ♢. According to the natural bidding system, we can see from the hands that the North player has 13 HCPs. Thus, 1D should be opened. When the teammate of the North player, the South player, receives this open-bid, he or she should understand the above information. Figure 10a shows the probability distribution of average HCPs of the North player predicted by the South player after getting the open-bid 1D. It is not difficult to see that the South player indicates that the probability of the North player’s HCPs is between 12–14, which conforms to the rules of the natural system.

Next, let us analyze the visualization results generated when the contract is finally obtained. Figure 10b shows that the West player has 0–2 ♠ cards, 6–7 ♡ cards, 0–2 ♢ cards and 3–5 ♣ cards, which is close to the real cards of the West player (2 ♠ cards, 6 ♡ card, 0 ♢ cards, 5 ♣ cards). Judging from the prediction results of the distribution of the West player (Figure 10c), there is a high probability of having a lot of ♡ cards. Furthermore, the West player might have the feasibility of a K ♡ card. In fact, the West player does own the K ♡ card. In brief, the above examples can prove that the visualization system can predict the information transmitted through the bidding and reasonably display hands understanding for players to communicate.

### 4.4. Comparison with State-of-the-Art

We compared the bridge bidding result with that in the work of [15]. We chose their different deep reinforcement learning models with layer = 2, 3, 4, 5, and ran the same 20,000, 140,000, 200,000 data. Since they studied the problem of bidding without competition, we provided a method based on the difference between the predicted IMP https://www.acbl.org/learn_page/how-to-play-bridge/how-to-keep-score/teams/ (accessed on 1 October 2020) and the best contracted IMP, which can be used to evaluate the bidding strength. Negative numbers indicate that the predicted results are inferior to the best results. The smaller the absolute value, the more accurate the prediction. The result in Table 3 demonstrates that our model has the best performance among all models, although our model is simpler without using reinforcement learning.

Meanwhile, we compare our bridge AI program with Wbridge5, the champions of the World Computer Bridge Championship 2016–2018. We can see that in Table 4, our program outperforms Wbridge5 by 0.41 IMP.

## 5. Conclusions

In this paper, we used the real bidding data provided by the Synrey platform to train the bidding model, which contains three-layer stacked RNN and multi-layer fully connected neural networks. Experiments showed that the accuracy of bid prediction reached 89%. At the same time, we have made a visualization system to intuitively explain the meaning of the bid, which is helpful to realize human–computer information interaction and make beginners learn the bidding rules better.

In future work, we would like to use reinforcement learning techniques to study the self-learning of bridge bidding. At present, there is still a big gap between bridge AIs and top human players. Nevertheless, we believe that subsequent research will better help bridge AIs achieve superhuman strength.

## Figures and Tables

**Table 1:** Parameters for BSM.

Type		Length
Input	The hands’ distribution of Player *i*: $X_{i}$	one-hot
	The bid in L round: $b_{L}$	(1*256)
	Vulnerable: v	(1*4)
	Is the player to bid: p	(1)
	check rules	(1*41)
Output	Bidding prediction	(1*41)
	The distribution of HCP: $P^{H}$	(1*38)
	The distribution of teammate’s hands: $P^{C}$	(4*13)
	The distribution of cards kept by players: $P^{Y}$	(3*52)

**Table 2:** **Test Case**. The actual situation is that the North and South players have nine cards with ♠, nine cards with ♢ and the East and West have 11 cards with ♡. The hands of both sides are very obvious. The North and South players prefer the contract called ♠ or ♢. It is biased towards the contract called ♡. After fierce bidding, the West player is the declarer, and the contract is 7H.

CardS	North	63.8.KJT932.AK63
	East	Q2.AJT92.Q854.JT
	South	AKJT985.6.A76.84
	West	74.KQ7543..Q9752
VUL	NS	
Bid Sequence	N:1D, 1H, 1S, 4H, P, P, 4S, 5H, P, P, 5S, 6H, P, P, 6S, 7H, P, P, X, P, P, P, END	
	Declare:West; The final contract:7H	

**Table 3:** Comparisons of the average difference of IMP between our model and the different models in [15].

Data Size	Our	[15] Layer = 2	[15] Layer = 3	[15] Layer = 4	[15] Layer = 5
20,000	−0.208	−2.213	−2.679	−5.213	−6.312
140,000	−0.163	−2.120	−2.558	−5.312	−6.136
200,000	−40.174	−1.986	−2.636	−5.232	−6.154

**Table 4:** Bidding strength comparision.

Model	Avg IMP	Max IMP	Min IMP
OURS-Wbridge5	0.41	0.8	−1.1

## Data Availability

Not applicable.
